# Supplementary figures and images for: Feedback from retinal ganglion cells to the inner retina
Source: PLoS One. 2021 Jul 22;16(7):e0254611. doi: 10.1371/journal.pone.0254611 (PMC8297895; doi:10.1371/journal.pone.0254611)

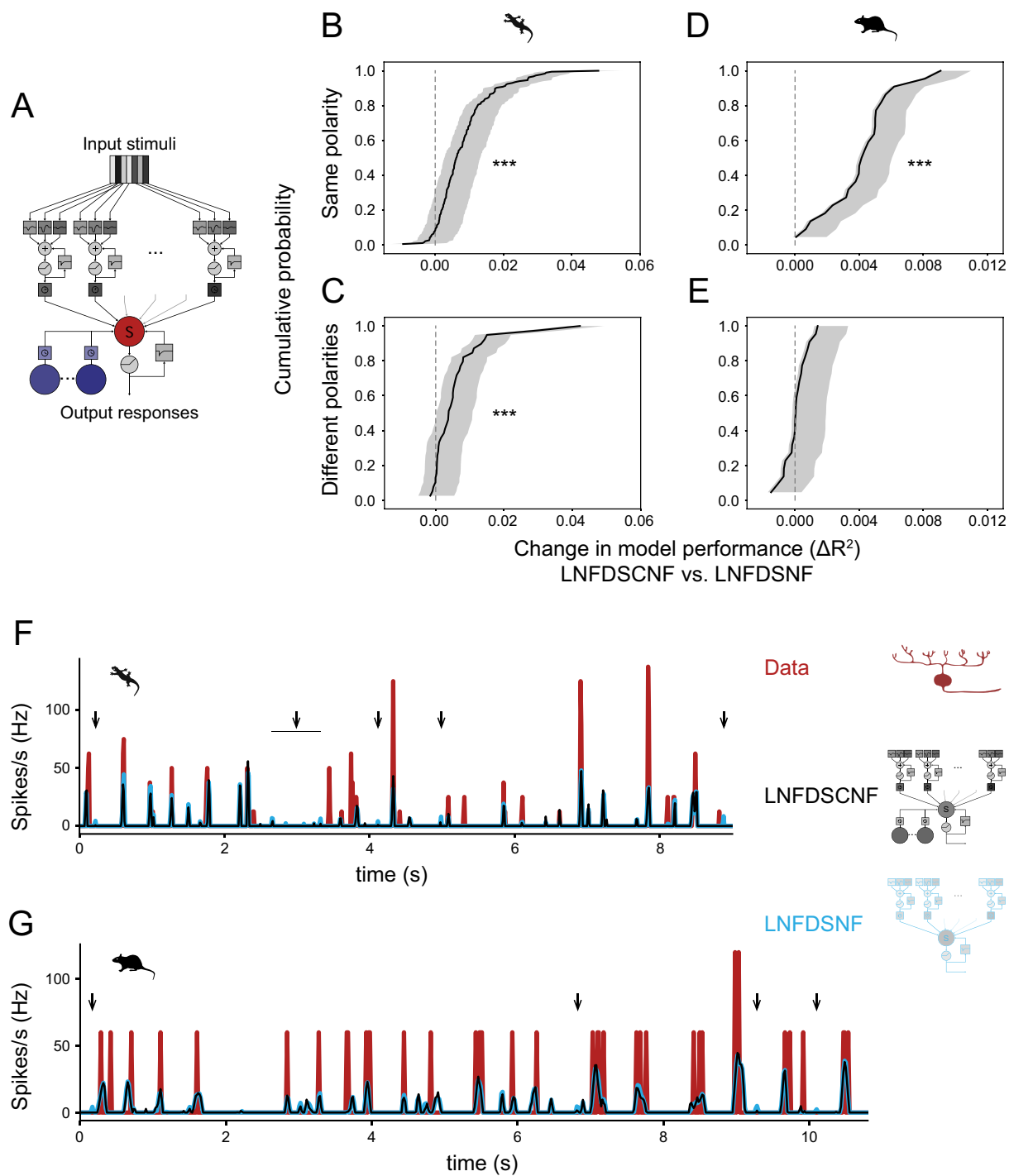

Supplement: S1 Fig — (A) Schematic diagram of the full circuit model with couplings (“LNFDSCNF” model). The model without couplings is identical to the “LNFDSNF” model in [30]. (B–E) Cumulative probability distributions (black; confidence intervals in gray) of LNFDSCNF model performance gain over LNFDSNF model for salamander (B, ΔR2 = 0.008±0.008 (mean ± standard deviation), p<0.001 (paired t-test), for coupled cells with the same response polarity; C, ΔR2 = 0.005±0.007, p<0.001, for those with different polarities; p = 0.054 from Kolmogorov-Smirnov test) and mouse (D, ΔR2 = 0.004±0.002, p<0.001; E, ΔR2 = 0.000±0.001, p = 0.4; p<0.001 from Kolmogorov-Smirnov test) retinal ganglion cells. The data are shown in the same format as Fig 2. (F, G) Time course of the firing rate (red) of representative salamander (F; cell #59 in Fig 1) and mouse (G) cells and that of the model outputs with (black, LNFDSCNF model) and without (cyan, LNFDSNF model) couplings. Arrows indicate the false-positive responses in the LNFDSNF model that were correctly suppressed in the LNFDSCNF model. (PDF) [file pone.0254611.s001.pdf]

**A**

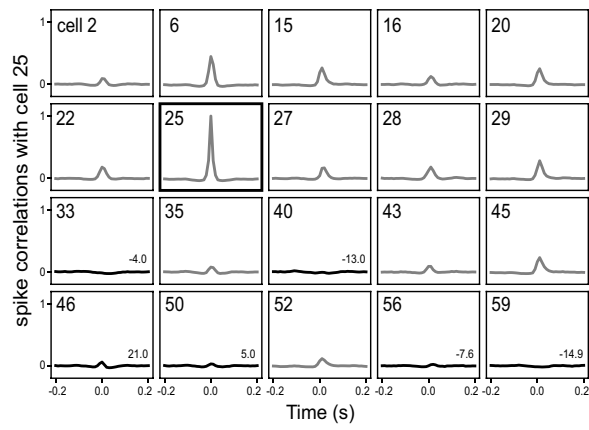

**B**

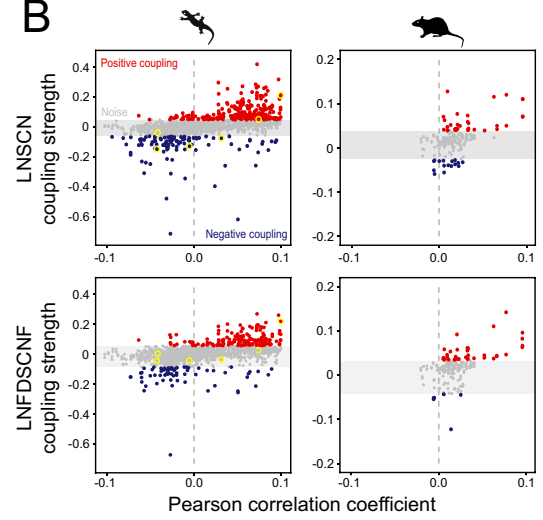

**C**

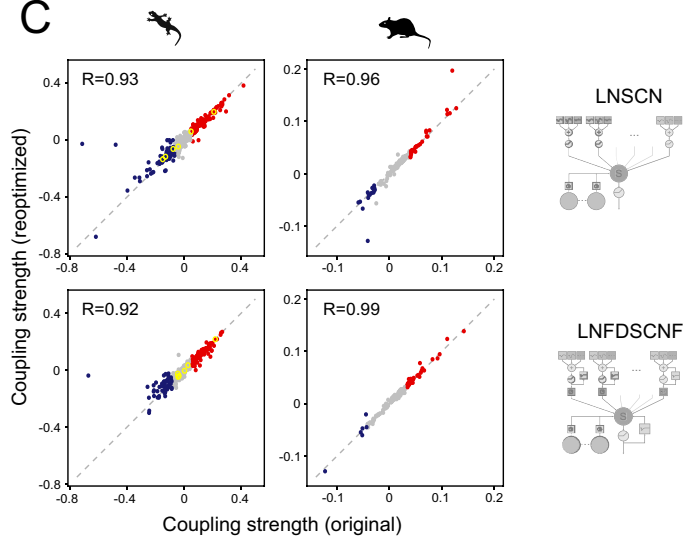

**D**

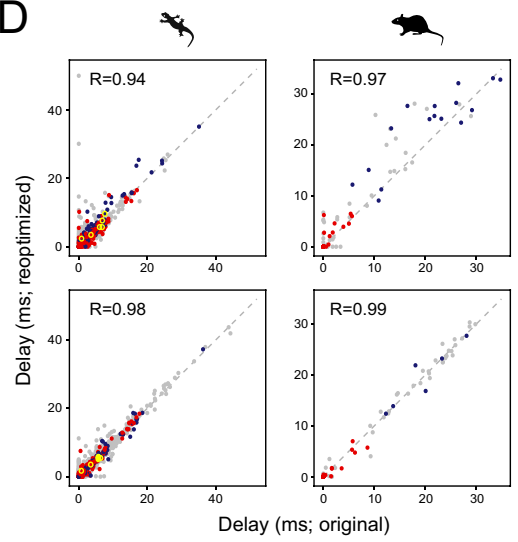

Supplement: S2 Fig — (A) Pairwise spike correlations of the representative cell (#25 from Fig 1B–1D; autocorrelation) and all the other simultaneously recorded 19 cells (cross-correlations). Those cells with too high correlations (gray; ≥0.1 or ≤−0.1 at the peak) were excluded from our model analysis to minimize the confounding effects of common visual inputs (see Methods for details). The coupling strength is shown on the bottom-right for each cell included in the LNSCN model targeting the cell #25 (black). (B) Coupling strength from the LNSCN (top) and LNFDSCNF (bottom) models as a function of the Pearson cross-correlation of firing patterns between ganglion cell pairs (left, salamander; right, mouse). Although we selected only those cells with low spike correlations (from −0.1 to 0.1) in the coupling models, we frequently found strong couplings in either polarity. The cells in the representative data set (from A) are highlighted with yellow circles. (C, D) Comparison of the model parameters (C, coupling strength; D, delay) between the original and reoptimized models after resetting them to be zero (top, LNSCN; bottom, LNFDSCNF; left, salamander; right, mouse). High Pearson cross-correlation values (R) in the perturbation analysis support a good convergence of the model fitting (see Methods for details). (PDF) [file pone.0254611.s002.pdf]

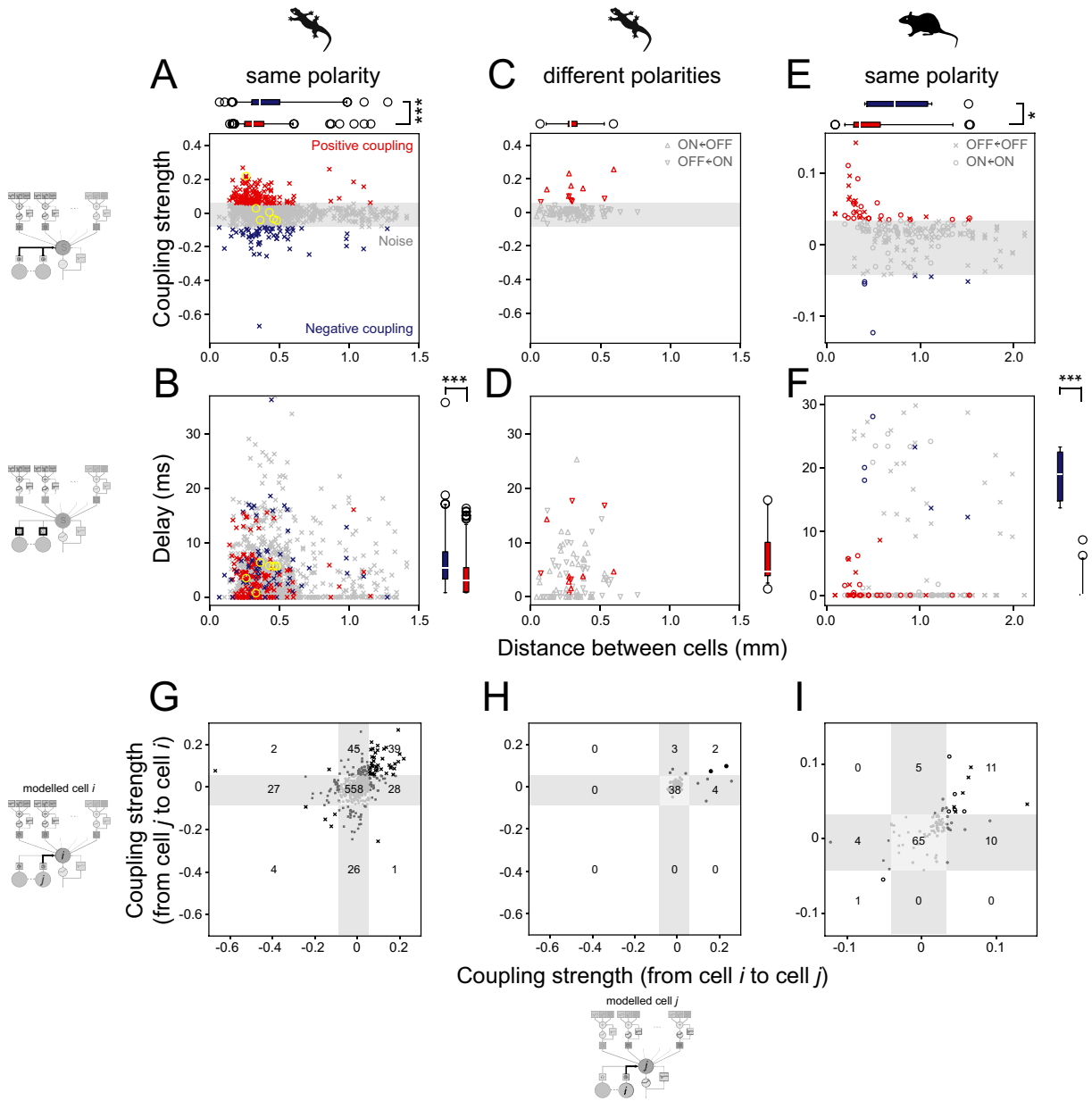

Supplement: S3 Fig — The data are shown in the same format as Fig 3 but for the LNFDSCNF model. (A, B) Coupling strength (A; ak in Eq (2) in Methods) and delay (B; lk in Eq (1) in Methods) parameter values plotted as a function of the distance between salamander retinal ganglion cells of the same response polarity. As is the case with the LNSCN model (Fig 3), positive couplings (red, N = 154) were found at a shorter distance (A; 0.31±0.14 mm versus 0.36±0.20 mm; median ± interquartile range (box plot); p<0.001, rank-sum test) and with a shorter latency (B; 2.3±4.7 versus 4.7±5.2 ms; p<0.001) than negative couplings (blue, N = 64). (C, D) Corresponding figure panels for salamander cells with different response polarities. Couplings above the noise level were all positive (N = 11) with the distance of 0.29±0.06 mm (C; median ± interquartile range) and the latency of 3.8±6.5 ms (D). (E, F) Corresponding figure panels for mouse retinal ganglion cells of the same response polarity. Likewise, positive couplings (red, N = 37) were found at a shorter distance (C; 0.35±0.28 mm versus 0.72±0.65 mm; p = 0.02) and with a shorter latency (D; 0.00±0.04 ms versus 19.1±7.7 ms; p<0.001) than negative couplings (blue, N = 6). Data not analyzed for the mouse cells with different response polarities because the coupled model performance did not improve significantly (S1E Fig). (G–I) Comparison of the coupling strengths from one cell to another and vice versa. Ganglion cells have symmetric couplings more frequently than expected in both salamander (G, p<0.001, χ2-test with df = 4, between cells of the same response polarity; H, p<0.001, between cells with different response polarities) and mouse (I; p<0.001, between cells of the same response polarity) retinas. The number of data points in each category is shown in the figure panels. (PDF) [file pone.0254611.s003.pdf]

**A**

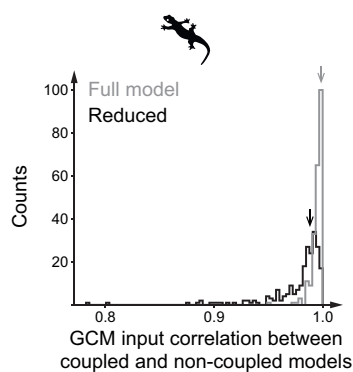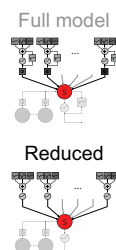

**D**

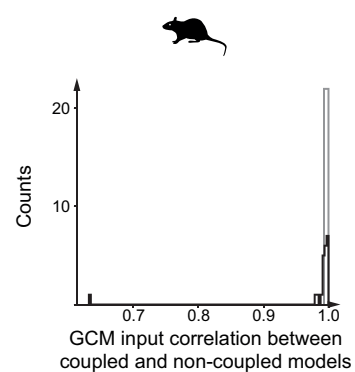

**B**

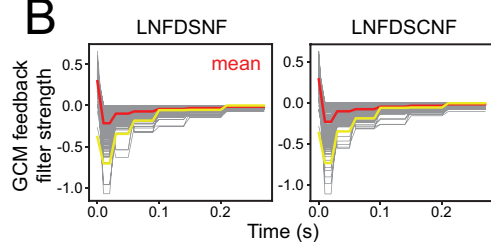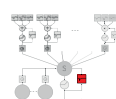

**E**

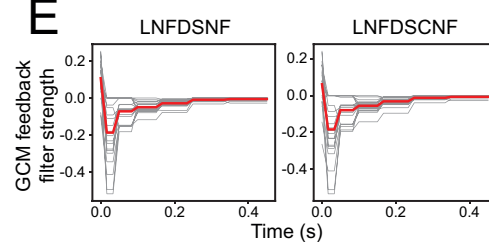

**C**

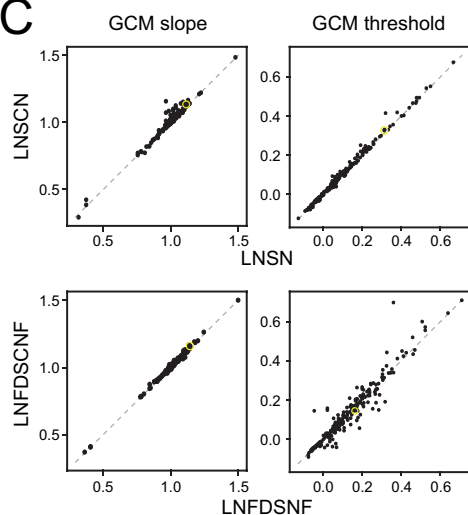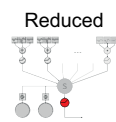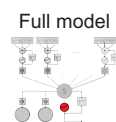

**F**

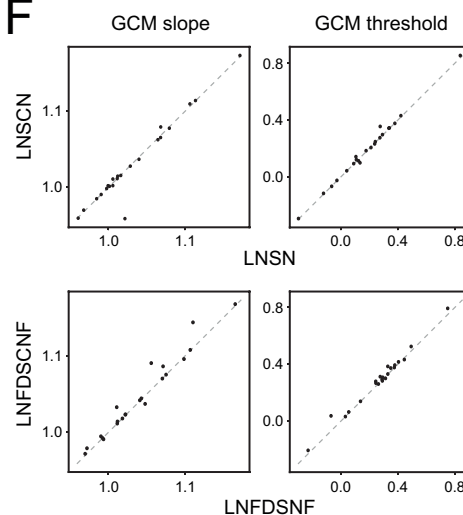

Supplement: S4 Fig — (A) Distribution of the ganglion cell module (GCM) input correlation between coupled and non-coupled models fitted to salamander cells (black, reduced models: LNSCN versus LNSN, 0.987±0.020, median ± interquartile range; gray, full models: LNFDSCNF versus LNFDSNF, 0.996±0.005). The correlation was calculated using the outputs of the summation (S) stage in the models (Eq (S9) in [30]). High correlations indicate that the GCM input dynamics are nearly identical in response to the visual stimuli (arrow, representative cell in Fig 1B–1D). (B) The second feedback (F) stage (GCM feedback filters; Eq (S7) in [30]) was nearly identical between the non-coupled (left, LNFDSNF) and the coupled (right, LNFDSCNF) models (yellow, representative cell in Fig 1B–1D; gray, all the other cells; red, mean). (C) Comparison of the second nonlinear (N) stage (GCM nonlinearity) between the coupled and non-coupled models (top, LNSN versus LNSCN; bottom, LNFDSNF versus LNFDSCNF; yellow, representative cell in Fig 1B–1D. Couplings did not affect the slope (left, α in Eq (S6’); Pearson’s R = 0.979 and 0.997 for the reduced and full models, respectively) or the threshold (right, θ in Eq (S6’); R = 0.955 and 0.997, respectively). (D–F) Corresponding figure panels for mouse retinal ganglion cells. There was no marked difference between the coupled and non-coupled models in GCM input correlations (D; black, reduced models, 0.994±0.005; gray, full models, 0.998±0.003), GCM feedback (E), GCM slope (F, left; R = 0.967 and 0.978 for the reduced (top) and full (bottom) models, respectively) or GCM slope (F, right; R = 0.991 and 0.996, respectively). (PDF) [file pone.0254611.s004.pdf]

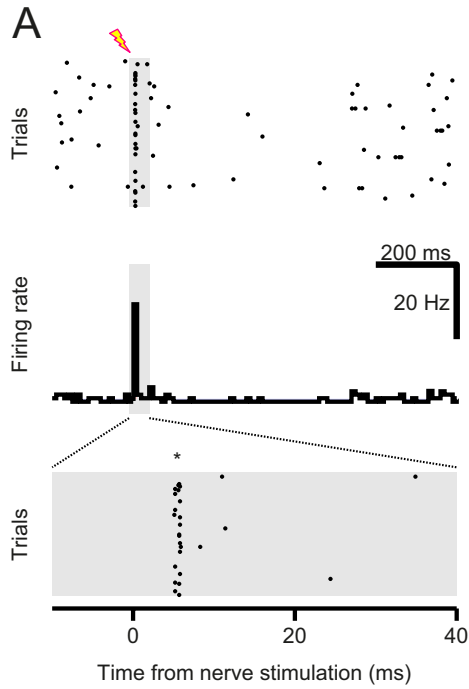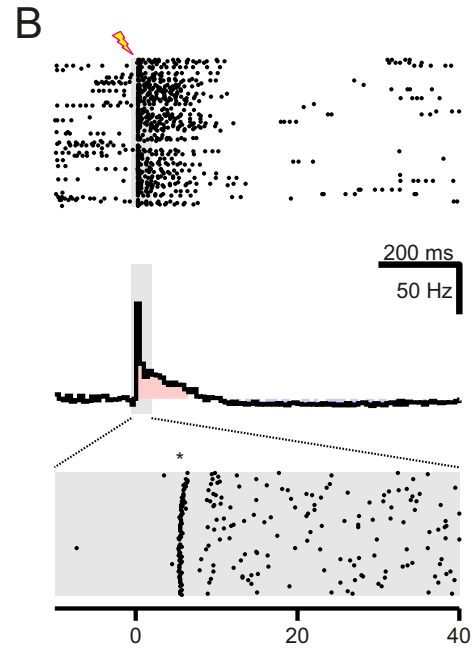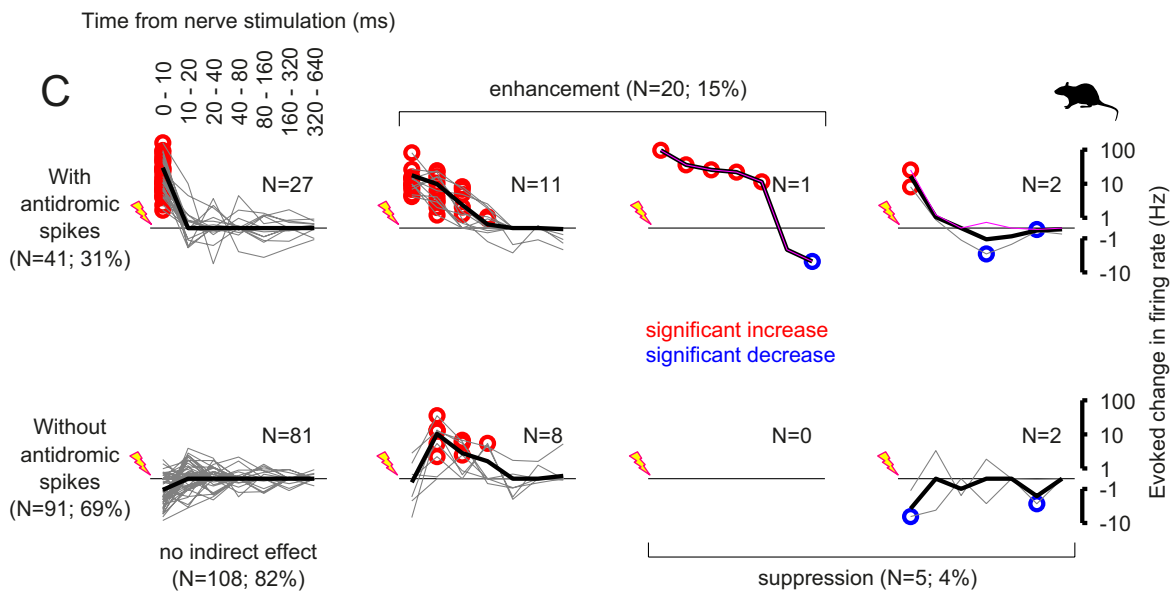

Supplement: S5 Fig — The data are shown in the same format as Figs 4 and 5 but for the mouse retina. (A, B) Firing patterns of two representative retinal ganglion cells, showing a period of suppression after the optic nerve stimulation (asterisk, antidromically evoked spikes) in the dark. Note the cell in B showed a period of enhanced firing before the suppression, while the one in A did not. (C) Population data of the ganglion cell responses to the nerve stimulation in the format of 2-by-4 contingency table, categorized by direct effects in rows and indirect effects in columns. Top row, cells with antidromic spikes; bottom row, cells without antidromic spikes. First column, no indirect effect; second and third columns, enhanced firing after the nerve stimulation (red circles, bins with significantly increased firing from the baseline); third and fourth columns, suppressed firing after the nerve stimulation (blue circles, significant decrease). (PDF) [file pone.0254611.s005.pdf]

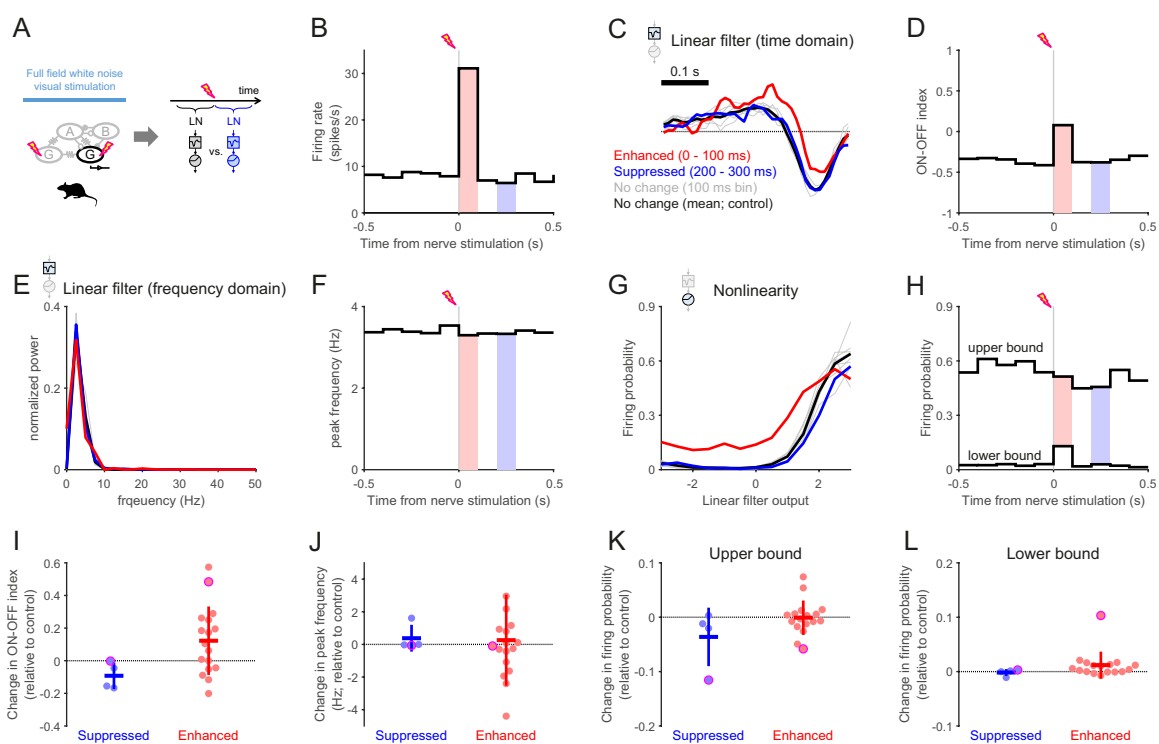

Supplement: S6 Fig — The data are shown in the same format as Fig 7 but for the mouse retina. (A) Schematic diagram of the experiment and analysis. (B) PSTH of a representative mouse ganglion cell with respect to the optic nerve stimulation during the visual stimulus presentation. Blue- and red-shaded bins indicate those with significantly higher and lower firing rates than the baseline, respectively. (C, D) Linear filters of the example cell (left) and corresponding ON-OFF indices (D; Eq (4) in Methods) at different time bins from the nerve stimulation. The filter became more biphasic during which the visual responses were enhanced by the nerve stimulation (red, 0–100 ms bin with significantly higher firing rates), but it went back to normal even when the visual responses were suppressed (blue, 200–300 ms bin with significantly lower firing rates; gray, all the other 100 ms bins, with the mean in black). (E, F) Normalized power spectral density (E) and the peak frequency (F; estimated by a Gaussian curve fit) of the linear filters in C. (G, H) Static nonlinearities of the example cell (G; Eq (5) in Methods) and the upper and lower bounds of the cell’s firing probability from sigmoidal curve fits (H; Eq (6) in Methods) at different time bins from the nerve stimulation. The upper bound was lower during the suppressed firing period (blue, 200–300 ms bin) while the lower bound was higher during the enhanced firing period (red, 0–100 ms bin) compared to the control period (gray, all the other 100 ms bins; black, mean). (I, J) Summary of the changes in the ON-OFF indices (I; suppressed, −0.09±0.08; enhanced, 0.12±0.21) and the peak frequencies (J; 0.38±0.83 and 0.26±2.29) between the periods with and without significant firing rate changes after the nerve stimulation (blue, decrease N = 4; red, increase N = 17; magenta, example cell in B–H). (K, L) Summary of the changes in the sigmoid function parameters fitted to the nonlinearity (K, upper bounds, −0.04±0.05 and −0.00±0.03; L, lower bounds [file pone.0254611.s006.pdf]
