# Supplementary material for: Feedback from retinal ganglion cells to the inner retina
Source: PLoS One. 2021 Jul 22;16(7):e0254611. doi: 10.1371/journal.pone.0254611 (PMC8297895; doi:10.1371/journal.pone.0254611)
Supplement: Attachment — Submitted filename: Response_to_reviewers.docx [file pone.0254611.s007.docx]

We thank the reviewers for their constructive comments. We have addressed all the concerns (in blue). Below are point-by-point replies (in black).

Reviewer #1: Review: Feedback from retinal ganglion cells to the inner retina

Summary

The authors use a combination of circuit modeling and experimental approaches to show that two distinct coupling pathways mediate local feedback from retinal ganglion cells in the retina. These two pathways consist of: (1) a fast and positive coupling between proximal ganglion cells and, (2) a slower negative coupling between distal ganglion cells via the connections with amacrine cells. These coupling pathways modulates the retinal ganglion cells’ response gain without affecting their feature selectivity.

The authors apply electrical perturbation of optic nerve and simultaneous use multi-electrode arrays to record the spiking activity from either salamander or mouse retinas. These recordings showed responses consistent with the existence of feedback circuity from retinal ganglion cells. Optic nerve stimulation triggered two distinct firing patterns of retinal ganglion cells: (1) a fast enhancement and (2) a slow, delayed suppression of spiking activity. Using different concentrations of potassium to modulate background firing rate they show that increasing spontaneous activity of retinal ganglion cells only affect the fast enhancement. This result supports the claim that the positive coupling is based on direct connections between retinal ganglion cells. The slow suppression could be eliminated by either blocking gap junctions or by blocking the inhibitory transmitters of amacrine cells. These results support the authors’ claim that the negative coupling pathway depends on the connections between retinal ganglion cells and amacrine cells. These findings are consistent with their modeling predictions.

Subsequently, to investigate how these coupling pathways affect the visual response properties of retinal ganglion cells, the authors record the ganglion cells’ visual response to a white-noise stimulus in combination with the optic nerve stimulation. The authors found that the ON-OFF index of each ganglion cell, which represent the feature selectivity remained largely unchanged. In contrast, the response gain is down-regulated during a period of suppression and up-regulated during a period of enhancement. These results support their claims that the feedback signaling pathway from ganglion cells could modulates the visual response gain but not the feature selectivity of retinal ganglion cells.

Taken together, we support publishing this paper with a major revision and some minor revisions stated below:

Major

First, the authors should add the comparison between the LNFDSNF model and the LNFDSCNF model. Figure 1D only shows that the LNSCN can explain the false-positive derived from the LNSN models. However, it is not clear whether this false positive could also be eliminated by just using the full model of LNFDSNF without considering the coupling parameter but only the feedback delay parameters. This needs to be clearly stipulated in the results section and ideally included in Figure 1D.

We totally agree that this is an important point, and clarified that the major difference between the coupled and non-coupled model outputs was in the false-positives even for the full model. Because adding more traces in Fig 1D made them illegible, we added new figure panels to Supplementary Figure 1 (S1 Fig) that compared the model outputs between the LNFDSNF and LNFDSCNF models. To support the generality of the claim, here we chose a different example for the salamander (revised S1F Fig; Cell #59 in Fig 1, not the cell 25 as in Fig 1D) and also showed an example from the mouse retina (revised S1G Fig). Thus, the suppression of false-positive responses by introducing the coupling term to the model was observed not just in one representative cell in our simple model framework, but also in many cells across species even with the full model framework.

See line 106 and S1 Fig legend in Line 819-823 in the revised manuscript.

Minor

Line 78-79: ‘~10s ms’ should be ~10 ms. ‘~100s ms’ should be ~100 ms.

In the revised manuscript (Line 45-46), we spelled them out for clarification: “for tens of milliseconds” and “for hundreds of milliseconds.”

Line 128-131: Delete ‘much’. The differences are that different from above where you use the word small.

We agreed and deleted the word “much” in the revised manuscript (Line 112).

Line 498 & Figure 3: The authors should clarify whether the model cell is an ON, OFF, or ON-OFF cell. The authors should also clarify the number of cells used in the analysis.

Different markers were used to indicate the cell types in the revised Fig 3 and S3 Fig. Specifically, to plot the coupling strength and delay, crosses and circles are used for OFF-to-OFF and ON-to-ON couplings, respectively (A, B, E, F: “same polarity”); and up- and down-pointing triangles for OFF-to-ON and ON-to-OFF couplings, respectively (C, D: “different polarity”). Similarly, to plot the reciprocity of the coupling strengths between cell pairs, crosses and circles are used for OFF cell pairs and ON cell pairs, respectively, in panel G and I (“same polarity”). In panel H (“different polarity”), however, each data point represents an ON-OFF cell pair. Thus, horizontal and vertical axes respectively represent ON-to-OFF and OFF-to-ON couplings for some data points, while OFF-to-ON and ON-to-OFF couplings for other data points. Here we decided not to use different markers because this would be 1) confusing; and 2) irrelevant to our claim that the connectivity is reciprocal.

The number of data points were described in the figure legend (panels A-F: Lines 791-811 in the original manuscript / Lines 145-164 in the revised manuscript), as well as in the revised figure panels G-I. Note that, for a given cell pair, there are two data points in panels A-F while only one data point in panels G-I. Thus, the total number of data points in G (N=730), for example, is half of that in A and B (N=1460).

Line 222 & Figure5A-B: The number of the ganglion cells used for data analysis is not clear. For example, the cells are separated into three groups: cells with only enhanced firing (23 cells), cells with only suppression of firing (13 cells), and cells with both enhanced firing and suppression (5 cells). And the total number is 34, how different cells are assigned to each group should be clarified.

Here we recorded 855 cells from 19 retinas in total. Of those, we selected cells that showed a spontaneous activity higher than 1 Hz for further analysis (control, 193 cells; high-potassium 324 cells). This selection of cells is critical to detect suppression. The figure panels in Fig 5A (control) are then organized as a 2-by-4 contingency table, as summarized in the table below:

|  | No indirect effect | Enhancement only | Both enhancement and suppression | Suppression only | Row total |
| --- | --- | --- | --- | --- | --- |
| With antidromic spikes | 34 | 23 | 13 | 5 | 75 (39%) |
| Without antidromic spikes | 76 | 19 | 4 | 19 | 118 (61%) |
| Column total | 110 (57%) | 42 | 17 | 24 | 193 cells |

The sum of the second and third columns gives the number of cells with enhanced firing (42+17=59 cells; 31%), and the sum of the third and fourth columns gives the cells with suppression (17+24 = 41 cells; 21%).

Fig 5B shows the data in the same format but with a high potassium condition, and S5C Fig shows the mouse data in the same format.

We described these numbers in the corresponding paragraph of the revised manuscript (Lines 267-281). For further clarification, we also put the number of cells along with the fraction in the revised Fig 5A,B and S5C Fig.

Line 244 & 253: The authors claim that blocking gap junctions ‘had little effect on the fast enhancement of the firing’ and blocking inhibitory transmission leave ‘the fast enhancement intact’. Is this conclusion based on the fraction of the cells with enhancement, or the firing activity of the same cell with enhancement in each condition? Please clarify in the result section.

The conclusion is based on the fraction of the cells with enhancement. The number of cells with enhancement did not change before and after applying the gap junction blocker applications (19 out of 19 cells; p<0.001, Fisher’s exact test; Fig 6B). Likewise, the number of cells with enhancement did not significantly change before and after blocking inhibitory transmission (13 out of 17 cells; p=0.02, Fisher’s exact test; Fig 6D). We thus concluded that these pharmacological treatments had little effect on the fast enhancement.

We clarified this point in the revised manuscript (Lines 291 and 301; see also the legend of Fig 6 in Lines 312 and 318).

Line 266: ‘fitted a linear-nonlinear (LN) cascade model to the visual responses of each cell’? The authors should explain which LN, LNSCN, or LNSN model is used here and why.

Here we used the LN model because a full-field stimulus was presented. The LNSN or LNSCN model requires spatial components. We clarified this in the revised manuscript (Lines 328 and 335-337).

Reviewer #2: In their paper, the authors investigate how RGCs can influence each other via gap junctions. To this end, they use data from salamander and mouse retina and first fit models to the data, which demonstrates statistical couplings between RGCs. They then stimulate the optic nerve to induce activity in RGCs, and find inhibitory slow effects, which they attribute to ACs based on pharmacological manipulation.

Overall, the paper is clearly written and appears technically correct. I have a few suggestions/remarks:7

1) Line 150: The authors claim that couplings were typically reciprocal, but I could not find a quantification of this finding.

The quantification was described in the figure legends (Fig 3G-I for salamander data: Lines 815-818 in the original manuscript / Lines 164-171 in the revised manuscript; S3G-I Fig for mouse data: Lines 954-958 in the original manuscript / Lines 853-858 in the revised manuscript). In short, the probability of having reciprocal couplings is significantly higher than the chance level, as opposed to that of having unidirectional couplings in both salamander and mouse data (p<0.001, chi-squared test with df=4). The number of cells in each category were shown in the revised Fig 3G-I and the S3G-I Fig.

The revised manuscript noted the quantification in the main text as well (Line 177).

2) Line 151: I don’t understand what evidence leads the authors to conclude that “single RGCs can be involved in multiple feedback pathways”.

Among the same polarity cells, we found that a large fraction of them received both positive and negative coupling signals (49 salamander and 12 mouse cells; 26 and 55%, respectively; see for example Fig 1C) as opposed to only positive (77 salamander and 6 mouse cells; 42 and 27%, respectively) or negative (9 salamander and 1 mouse cells; 5% each) couplings, or without significant couplings (50 salamander and 3 mouse cells; 27 and 14%, respectively.

| Number of cells (fraction) | Both positive and negative couplings | Only positive couplings | Only negative couplings | No significant couplings |
| --- | --- | --- | --- | --- |
| Salamander | 49 (26%) | 77 (42%) | 9 (5%) | 50 (27%) |
| Mouse | 12 (55%) | 6 (27%) | 1 (5%) | 3 (14%) |
| Column total | 61 (29%) | 83 (40%) | 10 (5%) | 53 (26%) |

Because the couplings are reciprocal (Fig 3G-I for salamander; S3G-I Fig for mouse), this means that the cells have reciprocal positive couplings with some cells (via gap junction syncytium), while reciprocal negative couplings with others. We thus concluded that “single RGCs can be involved in multiple feedback pathways.”

We clarified this point in the revised manuscript (Line 175).

3) Line 198ff: Can the authors estimate how many RGCs are stimulated by their optic nerve manipulation?

Yes, the number was given in Fig 5 and S5 Fig, the legends, and line 205 in the original manuscript. In short, about a third of RGCs showed antidromic spikes driven by the optic nerve stimulation: 75/193 cells (39%) in salamander retina under normal Ringer’s (Fig 5A, top row), 131/324 cells (40%) in salamander retina under high-potassium condition (Fig 5B, top row), and 41/132 cells (31%) in mouse retina in Ames’ medium (S5C Fig, top row).

We clarified these numbers in the revised manuscript (Lines 242 and 272).

4) Fig. 5A: The suppressive effects seem very small (I understand that they are hard to quantify). Even circles effectively showing zero effect are labeled significant. How was the test done? Is there some issue here?

We ran a shuffling analysis (10,000 repeats) to detect an enhancement or suppression for each cell at the significance level: alpha = 0.05 / 2 tail / 6 bins = 0.00416. Because the baseline firing rate is low in most cells (0.7 +/- 1.6 Hz; mean +/- standard deviation; our threshold is 1 Hz for the analysis), even a clear suppression effect (e.g., see a raster plot in Fig. 4C or S5A Fig) sometimes ended up with a small change in the firing rate. This is why even statistically significant suppression sometimes appears to be “effectively zero” in the log-scale PSTH (Fig 5). Note that, in many cases, significant suppression was observed across multiple bins. For better visualization and consistency across figures, we changed the cut-off firing rate in the log-scale PSTH from +/-0.75 Hz to +/-0.5 Hz in the revised Fig 5.

5) Line 215: Please show all data.

We performed negative control experiments in 3 salamander and 2 mouse retinas where we stimulated the bath without holding the optic nerve. And in all cases, we did not see any effects on RGC spike activity.

The revised manuscript included these data (Lines 250 and 607).

6) Lines 257 ff: I am surprised that the fitting for these very short periods after the nerve shock works so well. How many spikes went into this analysis for single cells in the respective periods?

Here we had 630 +/- 792 spikes (mean +/- standard deviation; N=160) in each time bin for the salamander cells, and 857 +/- 991 spikes (N=172) for the mouse cells. Note we ran 800-2,000 trials of nerve stimulation (with inter-stimulus intervals of 1-1.5 s) to compensate for a small bin size (0.1 s) and relatively low evoked firing rates (salamander, 3.5 +/- 3.0 Hz; mouse, 10.7 +/- 12.4 Hz; mean +/- standard deviation). For example, the representative cell in Fig 7B-H had the number of spikes ranging from 748 to 1037 in each time bin, and this was enough to fit an LN model. See Line 349 (the legend of Fig 7) in the revised manuscript.

7) Line 414: I am aware of an OFF bias in salamander recordings, but 185 to 4 seems excessive. What happened to the ON cells?

We identified 13 ON cells in 479 salamander cells (2.7 %). However, at least 2 simultaneously recorded cells should pass our selection criteria for the coupling model analysis. This ended up with only 4 cells (out of 189 cells; 2.1%) left for the modelling analysis (2 cells each from 2 recordings). See Line 485 in the revised manuscript.

8) Line 418: How does the selection of cells chosen by the authors ensure low false negatives?

Our spike sorting procedures occasionally returned more than one “unit” that had high cross-correlations and nearly identical spatio-temporal receptive field profiles. We considered that these “units” were likely the same cell but detected as different “units” in spike sorting by mistake because spikes from a cell can be picked up by multiple channels in our recordings. Among those “units”, the one with the highest number of spikes should have the lowest false negatives in spike detection, and this is why we chose it for further analysis while discarding the others. We clarified this point in the revised manuscript (Line 492).

Were these criteria determined before the analysis?

Yes, we determined those criteria before the modelling analysis (Line 488 in the revised manuscript).

How are the cells without clear LN model fit affected?

We have not analyzed those cells without clear LN model fit because the selection criteria were determined before the analysis.

While some RGC types (e.g., direction-selective ganglion cells) may not be responsive to the flickering bar stimulus, we expected that the cell types analyzed in this study (i.e., ON and OFF types) should respond robustly and their responses should be recapitulated well by the LN model (Baden, et al., 2019; Real, et al., 2017). Having no clear LN model fit indicates either 1) a cascade model framework is not appropriate for the cell in the first place, in which case a tailored model should be employed along with a tailored stimulus to probe its response properties; or 2) most spikes were not driven by the visual stimulus because, for example, the cell was unhealthy. This is why we discarded those cells.

9) Lines 461: What optimizer at which settings was used to fit the model? Is the code available online?

To fit free parameters of the model, we used the Truncated Newton algorithm (via “optimize.minimize” function from SciPy library) to minimize the objective function represented by the mean squared error between the measured firing rate and the model output (Lines 533-535 of the revised manuscript).

We are in the process of depositing the data and the analysis code online, and will make them available once the manuscript is accepted for publication.

10) Line 479: If the stimulus sequence during the test trials was identical to the training trials, the authors use a very weak notion of generalization. Could the authors also report generalization to data unseen during training? E.g. perform cross-validation with hold-out stimulus sequences.

The stimulus sequence was completely different between the training and test data sets in all cases, and we did cross-validation in our model analysis. For some data (from Real et al., 2017), we had a random stimulus sequence interleaved with a series of short identical sequences (i.e., B-A-C-A-D-A-...). We thus used the non-repeated part of the data (B,C,D,...; 5 min each) for training the model while the repeated part (A; 1 min) for testing the model. For the other data, we had a random stimulus sequence without any repeats, and thus took ~80% for training while ~20% for testing.

The revised manuscript clarified this point and inserted the proper citation (Line 550-554).

11) Line 566: Unclear which multiple comparison problem is being referred to. Consider ANOVA with post-hoc testing.

Here we referred to a multiple comparison problem with respect to the number of time bins we needed to test for the nerve stimulation effect on the spiking activity of individual cells. A linear bin size of 20 ms as in Fig. 4CD, for example, led to 50 bins for the 1 s time window. This will reduce statistical power (i.e., increase the false negatives) because running a statistical test for each bin would require a significance level of 0.05 / 2 tail / 50 bins = 0.0005 (with Bonferroni correction; note the “significance” in Fig. 4C were indicated without such correction for a display purpose). This is not ideal because detecting suppression is already difficult (due to low baseline firing rates). We thus instead took increasing bin sizes so that we had only 6 time bins to test for each cell. This allowed us to run a shuffling test at alpha = 0.05 / 2 tail / 6 bins = 0.00416.

We clarified this in the revised manuscript (Line 634).
